# Outgrowing self-denial

**DOI:** 10.7554/eLife.96286

**Published:** 2024-01-24

**Authors:** Jay K Goldberg

**Affiliations:** 1 Department of Ecology and Evolutionary Biology, University of Arizona Tucson United States

**Keywords:** sparks of change, research culture, being neurodivergent in academia, ADHD, mental health

## Abstract

After hitting rock bottom a few months into a prestigious fellowship, a postdoc recounts how they found their way to ADHD medication, therapy, and better mental health.

Kids most likely grow out of it. That’s what the doctors used to say to my mom after I was diagnosed with ADHD in the 1990s. I clung to these words after I started to refuse treatment as a teenager. I wanted to put behind me the years of feeling zombified by my medication and singled out by teachers, healthcare professionals and many of my peers. It would take me nearly 15 years to finally stop being in denial about my diagnosis and reconsider that decision – the end of a mental health journey that, in hindsight, I wish I had started much earlier.

It began with a phone call on an otherwise gloomy Midwest morning in 2020; the National Science Foundation was informing me that my proposal had been successful, and that it wouldn’t be long before I’d move to Tucson, Arizona, to start an independent postdoc. I was shocked… ecstatic even. Having my own ideas recognized in such a way pierced the low self-esteem I had accumulated after years of mistakes and failures stemming from untreated ADHD. Still wanting to believe that my diagnosis was a thing of the past, I saw these struggles as proof that I simply wasn’t good enough. Now, at last, I had a sign that I may actually deserve to belong in science.

I signed the contract as fast as I could – perhaps too fast, as I would come to understand much later. Eager to start my new project even in the middle of the pandemic, I relocated to Tucson soon after graduation, leaving behind my partner and my friends. I didn’t realize then that something else was about to disappear; the haphazard combination of coping mechanisms which I had been unconsciously using to manage my ADHD so far.

Of course, there was still caffeine and the likes, which I had always relied on to help me focus on monotonous tasks. But suddenly there were no opportunities for me to hide my feelings of self-loathing behind the curtains of friendship or socialization. Because of COVID, events where I could have met new people were sparse or non-existent (for good reason!) and I didn’t even meet most of my lab mates in person until roughly a year into my postdoc. With my dog and the local lizards as my only company, I became trapped in loneliness and crushing boredom.

Perhaps worse, I lost my most important coping strategy: science, which I stumbled upon when I changed majors to biology during my first year as an undergraduate. Until then I had found it nearly impossible to concentrate on my studies, with more stimulating thoughts grabbing my mind and leading me down curiosity-driven Wikipedia rabbit holes (in the best-case scenario). But biology and, later, research were different. They played nice with my scattered brain, allowing me the creative freedom to find answers by connecting ideas; not to mention the varied activities – field trips, lab experiments, computational analyses and, of course, reading and writing – that kept the days feeling new and exciting.

Due to the COVID-related constraints, however, it would take months before I could start the experiments for my project. Outside the lab, I was trying to learn my way around a new field that neither me nor my advisor had much experience with. The biological problems that fascinated me when writing my proposal suddenly all felt huge and insurmountable. I couldn’t make any progress, yet progress was essential since I was on a short-term contract. Worse, I felt I wasn’t living up to the expectations I placed on myself, let alone those of the National Science Foundation. Without science to keep my mind occupied, I began to look inwards instead. I realized that my low self-esteem had morphed into depression and I started to question whether I had, in fact, outgrown my ADHD.

Yet, in the end, these worrying thoughts weren’t enough to make me seek help. It took the shock of a traumatic encounter on a warm Arizona winter day for me to reach that tipping point. I had discovered a man’s lifeless body while hiking one of my local trails; I thought he was asleep, until I noticed the flies, that is. Struggling to process this event on my own, I finally called the campus psychological services helpline. Their response? “We can’t help you; you don’t have health insurance.”

Two months into my position, this is how I discovered that I hadn’t read the fine print when signing my contract. As a National Science Foundation postdoctoral fellow, I wasn’t recognized as an employee by my university, which meant I was on my own when it came to getting health coverage. Now I had also missed the deadline to register for student healthcare plans. I had finally acknowledged that I needed mental health support, only to realize that, due to my inattention, I had no access to healthcare at all. I had failed at life yet again.

This event triggered a downward spiral of mental self-flagellation and my brain started to play depressing thoughts on a loop while I stared at the ceiling of my casita.

*‘Other NSF postdocs read their contract and knew to purchase healthcare. Why wasn’t I smart enough to do the same?’*
*‘Why didn’t I pay more attention’?*
*‘I wouldn’t be in this mess if I didn’t have ADHD’.*

Hoping to find an answer, some magic explanation for why I was this way and how to shake it off, I turned to the scientific literature on ADHD. Maybe it was also a way to fill the void left by my inability to do the research I wanted. Sometimes, it was reassuring to find that others were like me, that most of what I hated about myself stemmed from a well-documented and treatable condition. Yet, at times, the idea that I would forever face uncurable neurodevelopmental problems made the helplessness overwhelming.

*‘My brain is broken and there’s nothing I can do.’*
*‘Why can’t I just be normal?’*

The memories of my past failures, which had been briefly kept at bay by the news of my fellowship, all came rushing back. One by one, I relived the experiences that had chipped away at my self-esteem – the teachers who told me I would never amount to anything, the classmates who thought I was stupid or annoying, the activities I wasn’t allowed to join, the bullying. I was unable to focus on anything else, not even the video games that had once captivated me. I considered quitting even though I had nothing else lined up, just to spite myself. Days turned into weeks that turned into months. The desert started to swelter under the summer heat.

In the end, the light came from senior colleagues who helped me gain access to my university’s healthcare services. The doctors’ first recommendation was an anti-depressant, and I filled in the prescription without any hesitation. Even though it didn’t help, I still held on to a sliver of optimism. I had hit rock bottom and was open to anything that might help me feel better.

It was my mother who suggested that perhaps I should reconsider ADHD medication. My memories of it were entirely negative, but in her recollection, it had done wonders for me as a child. She recalled moments I didn’t think could have happened, such as me sitting still to do my homework or teachers making positive comments on my behavior.

On day one of taking my pills, the thoughts that normally clouded my head felt calmer and quieter. For the first time in months, I actually paid attention to a Zoom meeting; I even got a little work done! I felt I had been put in control of my own brain again.

Still, I kept struggling with negative thoughts and self-loathing, especially in the evenings. After a while, I made the exciting yet frightening decision to start therapy. It took a few tries to find the right practitioner – I was once told that I was “obviously fine” since I had friends, a partner, and a PhD – but eventually, I got referred to Bob. Under his guidance, it finally dawned on me that my mistakes stemmed from manageable ADHD symptoms rather than irreparable personality flaws. I am no longer the poorly behaved child who people thought was stupid, lazy, unlikeable. I don’t have to keep on using these labels for myself.

Roughly two years later, I find myself more or less at peace with my neurodivergence. Bob’s help, combined with the medication, has allowed me to have new thoughts about myself, my past, and my feelings towards them. I understand and benefit from my ADHD drugs much more now that I take them based on how I feel and what I need, rather than because teachers wanted me to behave in a certain way. My mental health journey is far from over but reaching out for help and coming to terms with my neurodivergence were important steps along the way.

## About this article

This Sparks of Change article is part of a series of articles on being neurodivergent in academia, which includes a list of tips, resources and tools collated by neurodivergent scientists.

